# Factors associated with therapeutic response in acromegaly diagnosed in the elderly in Spain

**DOI:** 10.3389/fendo.2022.984877

**Published:** 2022-09-16

**Authors:** Betina Biagetti, Pedro Iglesias, Rocío Villar-Taibo, María-Dolores Moure, Miguel Paja, Marta Araujo-Castro, Jessica Ares, Cristina Álvarez-Escola, Almudena Vicente, Èlia Álvarez Guivernau, Iria Novoa-Testa, Fernando Guerrero Perez, Rosa Cámara, Beatriz Lecumberri, Carlos García Gómez, Ignacio Bernabéu, Laura Manjón, Sonia Gaztambide, Fernando Cordido, Susan M. Webb, Edelmiro Luis Menéndez-Torre, Juan J. Díez, Rafael Simó, Manel Puig-Domingo

**Affiliations:** ^1^Endocrinology Department, Diabetes and Metabolism Research Unit, Vall d’Hebron University Hospital and Vall d’Hebron Research Institute (VHIR), Universidad Autónoma de Barcelona, Barcelona, Spain; ^2^Department of Endocrinology, Hospital Universitario Puerta de Hierro Majadahonda, Madrid, Spain; ^3^Endocrinology Department, University Hospital of Santiago de Compostela, Neoplasia and Differentiation of Endocrine Cells Group, Instituto de Investigación Sanitaria-IDIS (Health Research Institute), Santiago de Compostela, Spain; ^4^Endocrinology Department, Cruces University Hospital, Biocruces Bizkaia, Endo-European Reference Networks (ERN), Barakaldo, Spain; ^5^Neoplasia and Differentiation of Endocrine Cells Group, Instituto de Investigación Sanitaria-IDIS (Health Research Institute), Santiago de Compostela, Spain; ^6^Department of Endocrinology, Hospital Universitario Basurto, Bilbao, Spain; ^7^Department of Endocrinology and Nutrition, Hospital Universitario Ramón y Cajal, Madrid, Spain; ^8^Department of Endocrinology and Nutrition, Hospital Universitario Central de Asturias, Asturias, Spain; ^9^Instituto de Investigación Sanitaria del Principado de Asturias (ISPA), Centro de Investigación Biomédica en Red Enfermedades Raras (CIBERER), Universidad de Oviedo, Oviedo, Spain; ^10^Department of Endocrinology, Hospital Universitario La Paz, Madrid, Spain; ^11^Department of Endocrinology and Nutrition, Hospital Universitario de Toledo, Toledo, Spain; ^12^Department of Endocrinology, Centro de Investigación Biomédica en Red de Enfermedades Raras (CIBERER), Hospital Sant Pau, Institut d’Investigacions Biomèdiques (IIB) SPau, Barcelona, Spain; ^13^Department of Medicine, Universitat Autónoma de Barcelona, Barcelona, Spain; ^14^Endocrinology and Nutrition Department, A Coruña University Hospital and A Coruña University, A Coruña, Spain; ^15^Department of Endocrinology, Hospital Universitari de Bellvitge, L’Hospitalet de Llobregat, Barcelona, Spain; ^16^Endocrinology and Nutrition Service, La Fe University Hospital, Valencia, Spain; ^17^Health Research Institute, Universidad País Vasco - Euskal Herriko Unibertsitatea (UPV-EHU), Centro de Investigación Biomédica en Red Enfermedades Raras (CIBERER), Centro de Investigación Biomédica en Red Diabetes y Enfermedades Metabólicas (CIBERDEM), Barakaldo, Spain; ^18^Department of Medicine, Instituto de Investigación Sanitaria Puerta de Hierro Segovia de Arana, Universidad Autónoma de Madrid, Majadahonda, Spain; ^19^Endocrinology and Nutrition Service, Germans Trias Hospital and Research Institute, Autonomous University of Barcelona, Badalona, Spain

**Keywords:** acromegaly, elderly, growth hormone, IGF-1 (insulin-like growth factor 1), somatostatin analog, surgery, Spain

## Abstract

**Context:**

Some reports suggest that acromegaly in elderly patients has a more benign clinical behavior and could have a better response to first-generation long-acting somatostatin receptor ligands (SRL). However, there is no specific therapeutic protocol for this special subgroup of patients.

**Objective:**

This study aimed at identifying predictors of response to SRL in elderly patients.

**Design:**

Multicentric retrospective nationwide study of patients diagnosed with acromegaly at or over the age of 65 years.

**Results:**

One-hundred and eighteen patients (34 men, 84 women, mean age at diagnosis 71.7 ± 5.4 years old) were included. Basal insulin-like growth factor type 1 (IGF-1) above the upper limit of normal (ULN) and growth hormone (GH) levels (mean ± SD) were 2.7 ± 1.4 and 11.0 ± 11.9 ng/ml, respectively. The mean maximal tumor diameter was 12.3 ± 6.4 mm, and up to 68.6% were macroadenoma. Seventy-two out of 118 patients (61.0%) underwent surgery as primary treatment. One-third of patients required first-line medical treatment due to a rejection of surgical treatment or non-suitability because of high surgical risk. After first-line surgery, 45/72 (63.9%) were in disease remission, and 16/34 (46.7%) of those treated with SRL had controlled disease. Patients with basal GH at diagnosis ≤6 ng/ml had lower IGF-1 levels and had smaller tumors, and more patients in this group reached control with SRL (72.7% vs. 33.3%; p < 0.04) [OR: 21.3, IC: 95% (2.4–91.1)], while male patients had a worse response [OR: 0.09, IC 95% (0.01–0.75)]. The predictive model curve obtained for SRL response showed an AUC of 0.82 CI (0.71–0.94).

**Conclusions:**

The most frequent phenotype in newly diagnosed acromegaly in the elderly includes small adenomas and moderately high IGF-1 levels. GH at diagnosis ≤6 ng/ml and female gender, but not age *per se*, were associated with a greater chance of response to SRL.

## Introduction

The behavior of GH-secreting tumors is heterogeneous and differs between patients (1–3). Although some evidence suggests that acromegaly could be less aggressive when diagnosed in elderly patients (4, 5), there are no age-adapted protocols and transsphenoidal surgery is also recommended as primary treatment in those patients (6–9). In addition, older patients are underrepresented in clinical trials due to eligibility criteria, resulting in scarce data on outcomes in this subset of the population (10–12). However, probably due to the increase in life expectancy, acromegaly is diagnosed more frequently than previously in elderly people and it is not expected to decrease (13, 14).

Elderly patients with acromegaly are also a heterogeneous group because some of them suffer from frailty and other age-dependent diseases such as cognitive impairment, which can add an additional challenge when deciding on treatment options (15). In fact, in the group of very old subjects (arbitrarily defined as those older than 75 years), the rate of patients’ rejection to surgery could be high, although data in this regard are scarce.

In order to gain further insights into the treatment options and patients’ response when acromegaly is diagnosed in elderly subjects, we have examined these questions in the real-world setting, performing a multicentric, nationwide, observational study of patients diagnosed with acromegaly at or over the age of 65 years.

## Patients and methods

This observational retrospective, multicentric, nationwide study ranged from 1995 to 2020 and included patients newly diagnosed with acromegaly at or over the age of 65 years. The study was endorsed by the Spanish Society of Endocrinology and Nutrition (SEEN) and was disseminated to all members of the Neuroendocrinology Task Force of the SEEN, which includes most of the endocrinologists who take care of acromegaly patients in Spain.

A specific registry was set up to collect real-life clinical features of acromegaly in the elderly and usual patients’ management modalities as well as the factors related to therapeutic response. A final version of the protocol was approved as a multicenter observational study with drugs by the Ethics Committees of the Vall d’Hebron University Hospital (number: PR(AG)318/2021). The study was conducted according to the mandates of the Declaration of Helsinki and good clinical practices. The patients’ confidential information was protected according to the Spanish data protection national law.

### Inclusion and exclusion criteria

Inclusion criteria were all patients with pituitary acromegaly diagnosed at or above the age of 65 years attending the different participating hospitals with at least 1 year of follow-up at the same hospital. Patients with incomplete data were excluded from the study.

### Variables and measurements

Clinical data, hormonal workup, imaging procedures, comorbidities, therapeutic modalities, and outcomes were recorded. For each patient, the following parameters were analyzed: year and age of diagnosis, gender, body mass index (BMI), specific clinical manifestations and comorbidities related to acromegaly, the estimated duration of symptoms before the diagnosis of acromegaly, and growth hormone (GH) and insulin-like growth factor type 1 (IGF- 1) levels. Regarding tumor features, sinus invasion evaluated by Knosp classification, sellar involvement, and chiasma compression were evaluated. Finally, we also evaluated first- and second-line therapies used for acromegaly, outcomes on efficacy and side effects of surgical and medical treatments, and disease activity at the last available visit of follow-up.

### Diagnosis of acromegaly and disease control

The diagnosis of acromegaly was made according to the clinical practice guideline criteria in force at the time of diagnosis. Incidental diagnosis was established when the diagnosis of acromegaly was made as a result of a cerebral MRI performed for any reason, which detected a pituitary adenoma, leading to the acromegaly diagnosis. Active disease was defined as IGF-1 level above 1.2 ULN. Controlled disease was defined when those patients under medical treatment presented IGF-1 levels within the specific age- and sex-adjusted reference range or <1.2 ULN. Cured or acromegaly in remission was defined when the IGF-1 level was normal without treatment, for more than 6 months.

Hormonal status was evaluated at diagnosis, after any treatment, and at the last visit.

### Statistical analysis

A descriptive analysis was performed. Numerical data are expressed as means and standard deviation (SD) (Gaussian distribution) or as medians (p50) and interquartile ranges (IQR) (non-Gaussian distribution). Categorical data are described using numbers and percentages. A comparison between two groups was performed using Student’s t-test or Mann–Whitney U-test for numerical data and chi-square test/Fisher exact test for categorical variables as appropriate. Median values across more than 2 groups [i.e., surgery vs. somatostatin receptor ligand (SRL) vs. dopamine agonist] were compared using the Kruskal–Wallis test with Bonferroni adjustment. We investigated which variables influence the response to SRL; predictors with p < 0.20 after Spearman’s rank test were included in multiple logistic regression, and a stepwise backward selection approach was used to identify the best predictive model. The model fit and calibration were assessed by Akaike’s and Bayesian information criterion, and the Hosmer–Lemeshow goodness-of-fit test, and the discrimination power by calculating the area under the receiver operating characteristic (ROC) curves (AUC). Finally, we formulated a predictive score useful for therapeutic decisions in clinical practice. Results were expressed as odds ratio (OR) and 95% CI. A two-sided P-value of <0.05 was considered significant. Statistical analyses were performed using the STATA16 statistical package (USA) for Windows.

## Results

### Patients’ characteristics: Biochemical and tumor features

Fifteen centers participated in the registry including patients diagnosed in the study period (1995–2020). From a total of 1,069 patients with acromegaly, 126 (11.8%) were older than 65 years at diagnosis. Among these 126 patients, two had an ectopic acromegaly and six were not included in the analyses due to incomplete data; therefore finally, 118 patients were analyzed.

Of the total, 84 (71.2%) were women and 34 (28.8%) men. The mean age at diagnosis was 71.7 ± 5.4 years without differences by gender. Patients with acromegaly diagnosed beyond the age of 75 years were also more frequently women (34.5% vs. 14.7%, P = 0.02) (**Table 1**).

**Table 1:** Baseline characteristics at diagnosis of acromegaly.

	All	Women	Men	P-value
N (%)	118	84 (71.2)	34 (21.8)	
Age at diagnosis, years (mean ± SD)	71.7 ± 5.4	72.3 ± 5.4	70.4 ± 5.2	0.09
Older than 75 years old n (%)	34 (28.8)	29 (34.5)	5 (14.7)	0.02
Older than 80 years old n (%)	9 (7.6%)	6 (7.1)	3 (8.8)	0.50
SBD, months (median, IQR)^a^	48.0 (100.0)	49.0 (101.0)	24.0 (96.0)	0.29
Incidental n (%)	20 (16.9)	12 (14.3)	8 (23.5)	0.29
GH, ng/mL (median, IQR)^a^	11.0 (11.9)	7.8 (11.2)	4.2 (7.2)	0.14
IGF-1, ng/mL (mean ± -SD)	610.1 ± 63.0	621.4 ± 60.3	582.9 ± 68.5	0.53
IGF-1 (ULN)	2.7 (1.4)	2.9 (1.5)	2.5 (1.0)	0.15
Diameter max, mm (mean ± -SD)^b^	12.3 ± 6.4	12.0 ± 6.6	12.7 ± 7.1	0.83
Macroadenoma n (%)	81 (68.6)	59 (70.2)	22 (64.7)	0.47
Extrasellar n (%)^b^	50 (47.6)	35 (48.0)	15 (46.9)	0.97
Chiasm compression n (%)	15 (14.3)	8 (11.0)	7 (21.9)	0.13
Sinus invasion n (%)^b^	49 (46.7)	34 (47.2)	15 (46.9)	0.71

SD, standard deviation; IQR, interquartile range; SBD, symptoms before diagnosis; GH, growth hormone; IGF-1, insulin-like growth factor 1; max, maximum. ^a^Reported in 110 patients. ^b^Reported in 105 patients.

The median time with specific symptoms before diagnosis (SBD) was 48.0 (100) [median (IQR)] months. In 20 (16.9%) patients, the diagnosis was incidental and the median time with SBD in this group was shorter [10 (85.0) months vs. 48 (100) (P < 0.02)] compared with patients in which the diagnosis was non-incidental. The maximal tumor diameter with accurate radiological tumor invasion description was available in 105 patients, and it was 12.3 ± 6.4 mm. GH and IGF-1 ULN were 11.0 (11.9) ng/ml and 2.7 ± 1.4 ULN, respectively, without gender differences (**Table 1**). Despite having relatively small tumors (the maximum diameter was 12.3 ± 6.4), 68.6% of patients had a macroadenoma.

Regarding the presence of acromegaly comorbidities, the most reported were hypertension in 91 cases (77.1%) and arthropathy in 54 (46.2%). Type 2 diabetes was only present in 40 (33.9%), which was not higher than the prevalence reported for acromegaly populations of younger age (16).

### First-line treatment options, complications, and side effects

**Figure 1:**
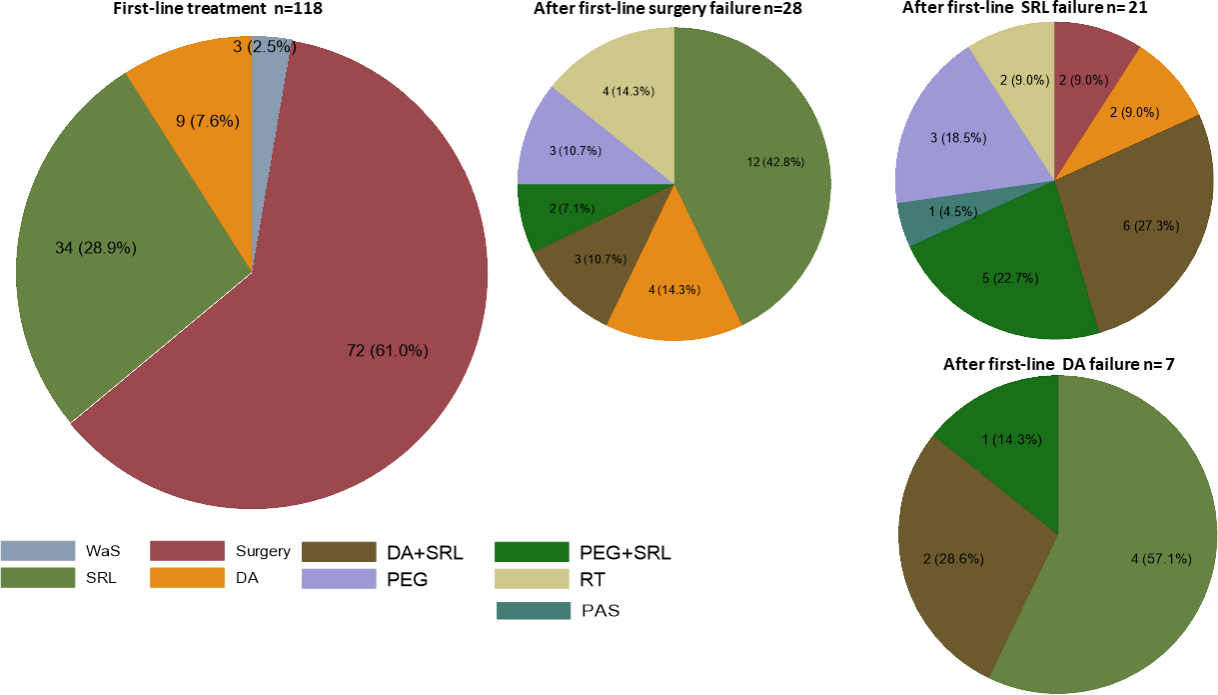
Treatment options. First-line on the left and second-line on the right. Results are expressed in “n” and percentage. WaS (wait and see) SRL, first generation somatostatin receptor ligand; DA, dopamine agonist ( all cabergoline); PEG, pegvisomant; PAS, pasireotide; RT, radiotherapy.

In total, 72 out of 118 (61.0%) underwent surgery. In 15 (12.7%) patients, surgery was contraindicated by the anesthetist or the pituitary care unit team, due to the high surgical risk for age and associated comorbidities, and 27 of the remaining 103 (26.2%) patients refused surgery. A total of 34 patients (28.9%) were treated with first-line SRL (50% with lanreotide and 50% with octreotide) and nine (7.6%) with dopamine analogues (DA), all of them with cabergoline (**Figure 1**); three patients refused any kind of treatment.

There was no postoperative mortality. Surgical complications were present in 25/63 (39.7%) of registered cases, with nine diabetes insipidus (four transient), three cerebrospinal fluid leakage, one III cranial nerve palsy, one epistaxis, two transient syndrome of inappropriate antidiuretic hormone secretion, and 17 new cases of hypopituitarism, and four cases had some subjective neurocognitive impairment. Regarding medical treatment, 13 out of 34 patients (38.2%) who were under first-line SRL experienced side effects: nine digestive, five mild hyperglycemia, two muscle pain, and four fatigue. However, in only three cases, the treatment had to be stopped due to side effects.

### Disease control and follow-up

After first-line surgery, 45/72 (63.9%) patients were in disease remission, and 16/34 (46.7%) of those treated with first-line SRL had controlled disease, without statistical differences between both treatment modalities.

In the follow-up, a second-line treatment was required in 57 (48.3%) patients, of which 28, 21, and seven received first-line treatment, surgery, SRL, or DA respectively. The most frequent treatment used as second-line was SRL alone or associated with DA (**Figure 1**). Radiotherapy was also indicated in six patients, four after surgery, and two after SRL.

**Figure 2:**
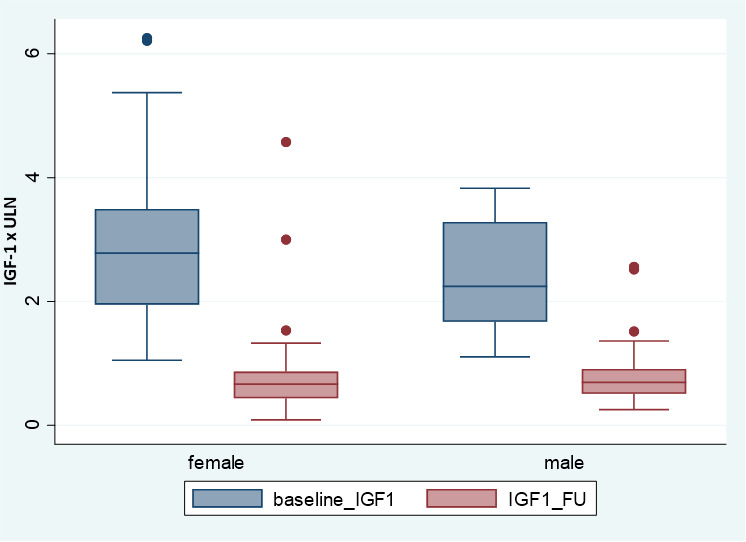
IGF-1 x ULN at baseline and in the last follow-up according gender.

The median of follow-up was 8.6 years [median (IQR) 103, (72.3) months]. At their last visit, 41/118 (34.8%) patients were in remission, 61/118 (51.7%) had controlled disease on medical treatment, and the remaining 16/118 (13.6%) had active non-controlled disease, five of them without medical treatment (three refused treatment after diagnosis and the other two refused any kind of treatment after experiencing side effects after medical therapy; all of them were patients older than 70). In those patients receiving active treatment, IGF-1 concentrations markedly decreased from baseline to the last visit, without gender differences (**Figure 2**).

### Disease characteristics in patients of or above the age of 75 years

A total of 34 out of 118 (28.8%) were patients ≥75 years old (**Table 2**); male gender was less frequently represented in these older patients [5 (14.7%) vs. 29 (85.3%); P < 0.02]. The median time with referred SBD was shorter in the older group (24.0 vs. 60.0 months; P < 0.01). Incidental diagnosis showed a statistical trend in those older >75 [9/34 (26.5%) vs. 11/84 (13.1%), P = 0.07; respectively]. Neither GH, IGF-1 ULN, tumor size, tumor invasion, nor percentage of comorbidities at diagnosis showed statistical differences when compared to those cases diagnosed between 65 and 75 years of age. Of these 34 patients aged 75 or older at diagnosis, only 12 (35.3%) underwent surgery, while 21 (61.8%) received medical treatment [16 (47.1%) SRL and five (14.7%) DA]. No statistical differences were found in the percentage of patients with controlled disease between both groups.

**Table 2:** Differences between patients older or younger than 75 years.

		≥75 years	65–75 years	P-value
N (%)	118	34 (28.8)	84 (71.2)	
Male gender	34 (28.8)	5 (14.7)	29 (34.5)	0.02
SBD, months (median, IQR)^a^	48.0 (100)	24.0 (50.0)	60.0 (99.0)	0.01
Incidental (n, %)	20 (16.9)	9 (26.5)	11 (13.1)	0.07
GH, ng/mL (median, IQR)^a^	11.0 (11.9)	12 (12.0)	10 (11.9)	0.50
IGF-1, ng/mL (mean ±- SD)	610.1 (63.0)	582.9 (68.5)	621.4 (60.3)	0.53
IGF-1 (ULN)	2.7 (1.4)	2.5 (1.0)	2.9 (1.5)	0.15
Diameter max, mm (mean ±- SD)^a^	12.3 ± 6.4	12.9 ± 6.4	12.4 ± 6.5	0.84
Macroadenoma n (%)	81 (68.6)	22 (64.7)	59 (70.2)	0.35
Extrasellar n (%)^b^	50 (47.6)	11 (36.7)	39 (52.0)	0.12
Chiasm compression n (%)	15 (14.3)	3 (10.0)	12 (15.8)	0.33
Sinus invasion n (%)^b^	49 (46.7)	16 (52.3)	33 (42.92)	0.22

SD, standard deviation; IQR, interquartile range; SBD, symptoms before diagnosis; GH, growth hormone; IGF-1, insulin-like growth factor 1; max, maximum. ^a^Reported in 110 patients. ^b^Reported in 105 patients.

### Clinical characteristics and therapeutic response according to basal GH levels

**Figure 3:**
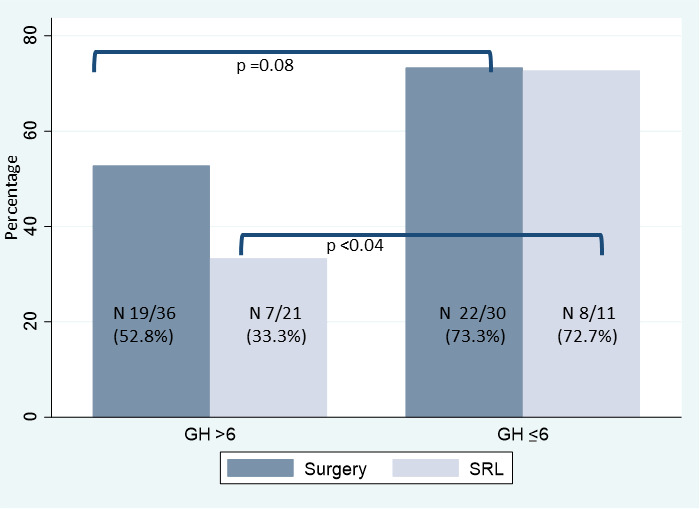
Percentage of patients controlled after surgery or SRL, according basal GH levels.

We evaluated if basal GH levels were related to disease control after SRL. For this, we assessed the optimal GH cutoff point with an ROC analysis. The binomial area under the receiver operating characteristic curve (AUC) with this unique variable was 0.72 (IC: 0.57–0.86), and the optimal cutoff was 6 ng/ml (sensibility: 85.20%, specificity: 63.80%, correct classification: 67.20%). Thus, we further divided the sample in patients according to GH levels. **Table 3** shows, age, gender, and tumor characteristics of both groups. The GH ≤6-ng/ml group included 49/110 (44.5%) patients; male gender was more frequent in this group [20 (40.8%) vs. 13 (21.3%) P < 0.02], and the time in months with referred SBD was shorter (24.0 vs. 60.0 months; P < 0.01), although incidental diagnosis was not more frequent. Regarding biologic and tumor features, the GH ≤6-ng/ml group had lower IGF-1 levels, as well as smaller and less extrasellar tumors (**Table 3**). The frequency of comorbidities at diagnosis was similar between the two groups. In the group with GH ≤6 ng/ml, more patients achieved disease control with SRL (72.7% vs. 33.3%; P < 0.04) and showed a statistical trend to a higher degree of surgical cure (73.3% vs. 52.8%; P = 0.08) (**Table 3** and **Figure 3**).

**Table 3:** Clinical characteristics and therapeutic response according to GH levels.

	All	GH ≤6 ng/dl	GH >6 ng/dl	P-value
N (%)	110	49	61	
Age (mean ±- SD)	71.6 ± 5.25	72.1 ± 5.8	71.4 ± 5.0	0.54
Male gender n (%)	33 (30.0)	20 (40.8)	13(21.3)	0.02
*BMI kg/m^2^* (mean ±- SD)	29.3 ± 4.9	30.9 ± 5.6	28.0 ± 3.9	0.01
SBD, months (median, IQR)^a^	48 (100)	24.0 (72.0)	60.0 (96.0)	0.01
Incidental n (%)^a^	20 (18.2)	7 (14.3)	13 (22.0)	0.33
IGF-1, ng/mL (mean ± SD)	586.2 ± 242.8	427.8 ± 170.8	674.0 ± 230.8	0.01
IGF-1 (ULN)	2.6 ± 1.1	1.9 ± 0.8	3.0 ± 1.1	0.01
Diam max, mm (mean ±- SD)^a^	12.3 ± 5.4	10.4 ± 4.2	13.3 ± 6.1	0.01
Macroadenoma	75 (68.2)	27 (55.1)	48 (78.7)	0.01
Extrasellar n (%)^b^	47 (44.7)	11 (28.2)	36 (50.7)	0.01
Surgery_remission n (%)	41/66 (62.1)	22/30 (77.3)	19/36 (52.8)	0.08
Controlled with SRL n (%)	15/32 (46.9)	8/11 (72.7)	7/21 (33.3)	0.04

SD, standard deviation; SBD, symptoms before diagnosis; GH, growth hormone; IGF-1, insulin-like growth factor 1; max, maximum. ^a^Reported in 110 patients. ^b^Reported in 105 patients.

### Predictive model of response to SRL

**Figure 4:**
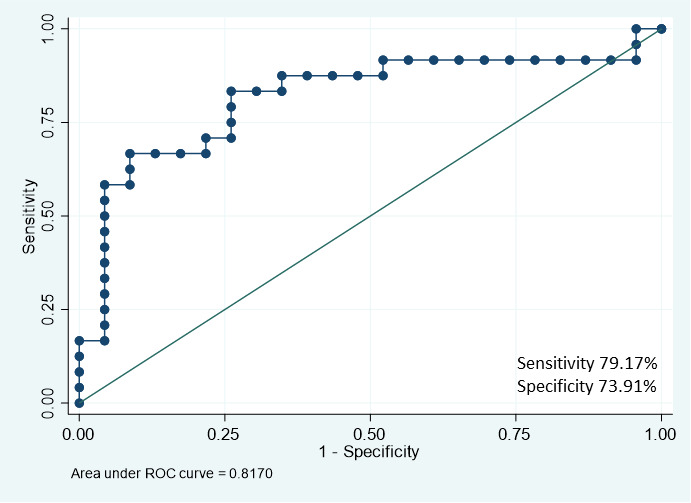
ROC curve for SRL response prediction performed according to the selected model.

We performed a stepwise backward selection approach to identify the best predictive model for response to SRL. The included variables were age at diagnosis, gender, basal GH, basal ULN_IGF-1, BMI, maximum tumor diameter, extrasellar extension, and type 2 diabetes. The reduced and parsimonious model that best explained SRL response included five predictors: basal GH, basal ULN_IGF-1, gender, maximum tumor diameter, and BMI. The model was well-fitted according to Akaike’s and Bayesian information criterion, and the likelihood ratio compared with the null model was <0.01. The binomial AUC was 0.82 CI (0.71–0.94) (**Figure 4** and **Supplementary Material**).

The odds ratio (OR) obtained for GH ≤6 ng/ml was 21.3 [IC: 95% (2.4–91.1)]. Thus, patients with GH ≤6 ng/ml were 21.3 times more likely to respond to SRL than those with GH >6 ng/ml, adjusted by (basal ULN_IGF-1, gender, maximum tumor diameter, and BMI). Male gender had a worse response with an OR of 0.09 [IC 95%:(0.01–0.75)], while the OR of SRL response for women was 11.11 [IC 95% (1.33–100)].

After log regression, with the predict command of the STATA package, we generated a new variable called “predictions” that estimated the probability of response to SRL in our sample. Then, a predictive table was obtained. For this, continuous variables were transformed into categorical variables (**Supplementary Material**). The results of the predictive table are shown in **Table 4**.

**Table 4:** Estimated probability of response to SRL expressed in percentage.

Gender and IGF (× ULN)						
Female				Male		
	IGF-1<1.5 × ULN	IGF-11.5-2.5 × ULN	IGF-1>2.5 × ULN	IGF-1<1.5 × ULN	IGF-11.5-2.5 × ULN	IGF-1>2.5 × ULN
**GH >6 ng/mL**	42.7	29.1	12.1	7.8	1.3	4.3
**GH ¾6 ng/mL**	92.0	86.1	67.0	56.7	43.0	23.0

SRL, somatostatin receptor ligand.

The table shows the estimated probability of response to SRL, expressed in percentage according gender IGF-1 and GH baseline levels.

## Discussion

Acromegaly is a chronic disease, which is diagnosed at any age, even in the elderly population. This is the first Spanish series comprising patients with acromegaly diagnosed at or over the age of 65 years. This subset represents up to 11.8% of the diagnoses of acromegaly in the present cohort in line with other registries (10, 11, 17–19). The median of time with SBD was 4 years (48.0 months), similar but slightly reduced than the one reported in the general Acromegaly Spanish Registry (20). This supports the idea that the reported increased incidence of acromegaly diagnosis in the elderly (11, 13, 14) is not a delay in the diagnosis. Probably, it may be the consequence of an increase in life expectancy, particularly in the Spanish population, although it could also be associated with a better awareness of the disease and in part with some degree of enhanced incidental diagnosis over recent years due to the extensive use of CT and MRI. However, the prevalence of incidental diagnosis in our series was virtually the same at 17% than the percentage recently reported by Giraldi et al. (21) in younger operated patients.

The disease characteristics of acromegaly diagnosed at older ages present some differences with those reported in the registries and large series (16) in younger patients, as exemplified in the present study. In our cohort, although up to 68% of the patients had a macroadenoma, the median of maximum tumor size was smaller than that reported in younger cases, with a smaller proportion consisting of invasive pituitary adenomas. These findings support the concept that in the elderly, GH-secreting tumors are less aggressive. However, biochemical data at diagnosis were similar to those previously reported in the Spanish Acromegaly Registry (22).

Therapeutic patterns in our series did not differ from general recommendation from guidelines, although in a subset of patients, surgery was contraindicated and in up to 26.2% surgery was refused, although postoperative mortality or serious peri-surgical morbidity was not high. Also, the percentage of patients controlled with first-line surgery was 63.9%, which did not differ from those controlled after first-line SRL (46.7%) and was not particularly different to what has been described for young adults (23). Four previous studies have described surgical outcomes in small cohorts of older patients with acromegaly (13, 24–26), all reporting surgical safety and biochemical efficacy in line with that reported for younger patients. However, there are no studies comparing first-line surgery vs. SRL specifically in elderly acromegaly patients in terms of efficacy, costs, and impact on comorbidities and on quality of life, a very important endpoint especially in this subset of patients. Thus, to increase the evidence regarding first-line treatment outcomes in aged patients, it is important to perform such study in the near future, especially considering the longevity of the general population.

Our results suggest that GH at diagnosis is a strong predictor for SRL response in aged people suffering from acromegaly, particularly in women. As far as we know, this is one of the few studies which analyze predictive factors implicated in the therapeutic response in older acromegaly patients. In our work, neither the age (using an arbitrary cutoff of 75 years old) changed acromegaly clinical presentation in terms of tumor size or GH/IGF-1 circulating concentrations, nor was the overall response to treatment different at very advanced ages. Of note, first-line medical therapy, achieved as good control as first-line surgery, thus reinforced the efficacy and safety of medical treatment in this particular group of patients. In this regard, we found that patients with GH lower than 6 ng/ml at diagnosis had a milder acromegaly phenotype, not just biochemically but also regarding tumor burden (smaller and less extrasellar tumor), and more patients achieve disease control with SRL (27). However, differently than what has been reported by others (28, 29), basal GH levels at diagnosis were not predictive of surgical remission in our series; our data may be in part explained by the fact that surgical treatment was performed by many different neurosurgical teams as the series consisted of patients from all major pituitary centers in Spain. Previous studies showed a correlation between tumor size and mean GH levels (30, 31). This could suggest that a smaller tumor and a lower GH at the time of diagnosis identify patients at an earlier stage of the acromegaly natural history, or alternatively, that their tumor is *per se* of low grade. However, most of these studies found that in the group with lower GH the patients were older (31–33). Likewise, in the study of Espinosa de los Monteros et al. (31), the frequency of symptoms, signs, and comorbidities was similar between GH groups, against the concept that low GH levels represent simply an early acromegaly disease detection. Similar to this study (31), in our cohort male gender was more frequently represented in the GH <6-ng/ml group in contrast with previously published series (31–33). In the low GH <6-ng/dl group, a higher percentage of patients achieved disease control with SRL compared to patients with higher basal GH, thus reinforcing the concept that acromegaly is a heterogeneous disease even at older ages and that the disease characteristics *per se* and not age are what determine clinical behavior and therapeutic response.

In the present study, basal GH levels alone had a remarkable predictive power for SRL response with an AUC of [0.72 IC: (0.57–0.86)] increasing up to 0.82 CI (0.71–0.94) when some variables that are usually available when acromegaly is diagnosed are added. The predictive model allowed the formulation of a score for clinical application in which GH and gender were the most powerful predictors for SRL response, adjusted by IGF-1 level, tumor size, and BMI.

Some other studies have also reported the response to SRL in older patients. The *post-hoc* analysis of the PRYMARYS clinical trial (34) examined 90 treatment-naïve acromegaly patients with macroadenomas, the potential predictive factor to SRL response, and found that older age, female sex, and lower IGF-1 levels at baseline were associated with an increased probability of achieving long-term hormonal control. As in our study, female sex and IGF-1 were good predictors.

Coopmans et al. (35) analyzed 622 acromegaly patients from two cohorts, 194 from Rotterdam and 428 from the Liège Acromegaly Survey Cohort treated initially with maximum doses of SRL. The baseline IGF-1 concentration was the best predictor of good biochemical response to SRL, followed by BMI. Younger age at diagnosis appears to be the most important determinant of not responding, and a significant inverse association was seen between GH concentration at diagnosis and absolute IGF-1 reduction after treatment. Recently, Nista et al. (36) retrospectively evaluated the potential predictors of SRL response in a cohort of 55 naïve acromegaly patients aged 57 (IQR: 45–63) and found that dichotomized age, IGF1 ULN at diagnosis, and T2-hypointense MRI signal of the tumor were reliable predictors of SRL response. However, this study was not fully comparable to ours, as patients were much younger.

In our cohort of elderly patients, low basal GH and female gender were the most powerful predictors of SRL response. IGF-1, BMI, and tumor size also contributed with less etiologic fraction and were adjusting factors. Regarding tumor size, in our cases the presence of a macroadenoma showed a statistical trend for good response, although it should be noted that, despite having up to 60% of macroadenomas, the mean tumor size was only of 12.3 ± 6.4 mm (only four patients had tumors larger than 25 mm), and invasiveness was low in our cohort. The knowledge of these results will allow physicians to better advise their elderly patients when they ask about surgery vs. SRL response, especially in the subgroup of patients that refuse or have contraindication for having surgery, thus helping to share the decision-making process.

Our study has some limitations: it is of retrospective nature and therefore prone to introduce some bias, as patients were recruited in different centers in which clinical practice is based on clinical judgement according to clinical guidelines and a predefined protocol was therefore not used. GH (and IGF-1) assays had been modified (and eventually improved) in the time period analyzed. The strength of this study lies in the number of patients included, since it is among the largest studies reported with a nationwide distribution. In addition, the analyses performed used clinical data allowing comparability and usefulness for clinical practice.

In conclusion, the most frequent phenotype in elderly people with acromegaly includes relatively small adenomas and moderately high IGF-1 levels. GH at diagnosis ≤6 ng/ml and female gender, but not age *per se*, were associated with greater chance of response to SRL, thus confirming the heterogeneous nature of the disease, which also applies to patients aged 65 or older.

## Data availability statement

Authors agree to make data and materials supporting the results or analyses presented in their paper available upon reasonable request.

## Ethics statement

The studies involving human participants were reviewed and approved by ethics committees of the Vall d’Hebron University Hospital (number: PR(AG)318/2021). The ethics committee waived the requirement of written informed consent for participation.

## Author contributions

Conceptualization, BB, MP-D; methodology, BB; writing—original draft preparation, BB. writing—review and supervision, MP-D All the authors participated in the data collection and contributed to the article and approved the submitted version.

## Conflict of interest

BB, MP, AV, CA-E, AV, RC, IB, SG, FC, SW, and MP-D have received consulting fees or research support or participated in clinical trials supported by from Pfizer, Ipsen, Quiasma, and/or Recordati.

The remaining authors declare that the research was conducted in the absence of any commercial or financial relationships that could be construed as a potential conflict of interest.

## Publisher’s note

All claims expressed in this article are solely those of the authors and do not necessarily represent those of their affiliated organizations, or those of the publisher, the editors and the reviewers. Any product that may be evaluated in this article, or claim that may be made by its manufacturer, is not guaranteed or endorsed by the publisher.
