# Supplementary material for: Factors associated with therapeutic response in acromegaly diagnosed in the elderly in Spain
Source: Front Endocrinol (Lausanne). 2022 Sep 16;13:984877. doi: 10.3389/fendo.2022.984877 (PMC9523598; doi:10.3389/fendo.2022.984877)
Supplement: Supplementary file 1 [file DataSheet_1.pdf]

Supplementary material

Multivariable analysis to predict SRL response

	OR	OR 95% CI	p
GH ≤ 6ng/ml	21.26	2.37 - 91.08	0.006
Male-Gender	0.09	0.01 - 0.75	0.026
IGF-1 categorized			
1	0.15	0.01 - 4.10	0.262
2	0.83	0.05 - 14.40	0.900
Macroadenoma	5.18	0.62 - 42.48	0.127
BMI categorized			
Overweight	0.63	0.07 - 5.46	0.675
Obesity	1.73	0.17 - 17.86	0.645

The final model to predict SRL response included: basal GH, basal ULN_IGF-1, gender, maximum tumour diameter and BMI. CI: confidence intervals, GH: growth hormone, IGF-1 insulin-like growth factor, BMI: body mass index.

After log regression, with the predict command of STATA package, we generated a new variable called "probability" that quantified the probability of SRL good response in our sample. Then a predictive table was generated. For this continue variables were transformed into categorical as follow: (**Basal GH**: GH < 6ng/ml: 1 and GH ≥ 6ng/ml: 0 , **Basal ULN_IGF-1**: (<1.5: 0, 1.5-2.5: 1 and >2.5: 2), **maximum tumor diameter**: (<10mm: 0 ; ≥ 10mm: 1) and **BMI:** (< 25Kg/m^2: 0 "normal", 25-29.9 Kg/m^2: 1 "overweight" and >29.9 Kgm^2=2 "obesity").

Below are all the predictive table combinations expressed in percentage of estimated SRL good response

Table 4a: Estimated probability of response to SRL expressed in percentage taking into account basal GH levels and gender

	Female	Male
**GH>6**	28.24	4.47
**GH<6**	81.73	40.00

Table 4b: Estimated probability of response to SRL expressed in percentage tacking into account basal GH levels, gender and basal levels of IGF-1

	Gender and IGF					
	**Female**			**Male**		
	0	1	2	0	1	2
**GH>6**	42.73	29.10	12.10	7.79	1.30	4.30
**GH<6**	92.02	86.16	67.00	56.69	43.04	23.01

0: IGF-1 <1.5, 1: IGF-1 (1.5.2.5) and 3 IGF-1 >2.5ULN

Table 4c: Estimated probability of response to SRL expressed in percentage taking into account basal GH levels, gender and maximum tumor diameter

		Female	Male
**Micro**	**GH>6**	14.10	1.55
	**GH<6**	71.54	23.35
**Macro**	**GH>6**	42.39	7.38
	**GH<6**	91.92	56.66

Table 4d: Estimated probability of response toSRL expressed in percentage taking into account basal GH levels, gender, IGF-1 and maximum tumor diameter

		Gender and IGF					
		**Female**			**Male**		
	**IGF-1**	0	1	2	0	1	2
**MICRO**							
	**GH>6**	24.27	0.10	4.85	2.76	0.41	1.88
	**GH<6**	87.37	48.24	78.62	35.40	7.97	27.55
**MACRO**							
	**GH>6**	63.42	21.31	51.11	12.81	2.27	9.69
	**GH<6**	96.94	83.09	95.89	77.00	37.96	64.53

0: IGF-1 <1.5, 1: IGF-1 (1.5.2.5) and 3 IGF-1 >2.5ULN

Table 4e: Estimated probability of response to SRL expressed in percentage taking into account all the adjusting variables

Tumor size<br>BMI<br>GH	Gender and IGF-1					
	------------- Female ----------			------------- Male ------------		
	**0**	**1**	**2**	**0**	**1**	**2**
**MICRO**						
**Normal weight**						
**GH>6**	23.75			2.76		
**GH<6**	86.88			37.64		
**MICRO**						
**Overweight**						
**GH>6**		14.02			1.46	
**GH<6**		77.61			24.01	
**MICRO**						
**Obesity**						
**GH>6**			4.51			0.43
**GH<6**			50.12			8.39
**MACRO**						
**Normal weight**						
**GH>6**	61.72			12.81		
**GH<6**	97.17			75.76		
**MACRO**						
**Overweight**						
**GH>6**		45.78			7.15	
**GH<6**		94.72			62.06	

**MACRO**						
**Obesity**						
**GH>6**			19.66			2.18
**GH<6**			83.88			32.17
